# Advances in Neurodegenerative Diseases

**DOI:** 10.3390/jcm12051709

**Published:** 2023-02-21

**Authors:** Jeroen Van Schependom, Miguel D’haeseleer

**Affiliations:** 1 Center for Neurosciences, NEUR and AIMS Research Groups, Vrije Universiteit Brussel (VUB), Laarbeeklaan 103, 1090 Brussel, Belgium; 2 Department of Electronics and Informatics (ETRO), Vrije Universiteit Brussel, Pleinlaan 2, 1050 Brussels, Belgium; 3 Department of Neurology, Universitair Ziekenhuis Brussel (UZ Brussel), Laarbeeklaan 101, 1090 Brussels, Belgium; 4 Nationaal Multiple Sclerose Centrum, Vereeckenstraat 44, 1820 Melsbroek, Belgium

Neurological disorders are the leading cause of physical and cognitive disability across the globe, currently affecting approximately 15% of the worldwide population [1]. Absolute patient numbers have considerably climbed over the past 30 years. On top of that, the burden of chronic neurodegenerative conditions is expected to at least double over the next two decades. Because of this evolution, which can largely be attributed to the expansion of the aging population, it will be a huge challenge to keep neurological care accessible to everyone. Alluding to such threats, leading institutions such as the World Health Organization and the United Kingdom’s National Health Service have already dropped the alarming quotes that “available resources for neurological services are insufficient in most countries of the world compared with global need for neurological care” and that “neurological services are not sustainable in their current form and redesign is needed” [2].

In addition to the ‘direct’ costs associated with the (para)medical management of neurodegenerative disorders, it is important to realize that the total financial impact, including expenses related to reduced quality of life and/or employment, may stretch to even greater proportions. This point is particularly relevant in frequently occurring and debilitating conditions that span several decades of patient-years, such as multiple sclerosis (MS) and Alzheimer’s disease (AD). For example, it is estimated that up to 80% of the subjects affected by MS become unemployed within the first 15 years following diagnosis, leading to a cost that is about 4000 USD per year higher compared to healthy age- and gender-matched controls [3], whereas the majority of the yearly total patient care expenses in AD (approximately USD 300 billion) is used to cover for institutionalization [4,5].

This Special Issue is dedicated to the standpoint that next-generation research will need to push the current knowledge boundaries of neurology in a multi-level effort to overcome the abovementioned concerns. Research aimed at (a) improving pathophysiological understanding, (b) outlining new strategies for disease prevention, modification, and curation, (c) developing novel biomarkers that enable early diagnosis, the detection of (subclinical) disease progression, and/or treatment response monitoring, and (d) facilitating care delivery in chronic neurodegenerative disorders is essential to achieve our mission. The combination of these strategies is expected to prevent and/or at least delay neurological decline in a large number of individuals, will likely release the pressure from the available logistic and financial resources, and should modernize health care for people with such disorders. Bravery, out-of-the-box thinking, and creativity may all prove to be valuable assets along the way, so we would like to specifically encourage studies exploring innovative hypotheses arising from solid conceptual frameworks but not necessarily grounded by existing paradigms, concepts, and/or customs.

MS is a leading cause of chronic non-traumatic disability in young adults and can therefore serve as an appropriate illustrative model. The first fundamental building block towards treating any condition is understanding how the disease originates and how it progresses. Examples of recent paradigm shifts, in contrast to older beliefs, in the pathophysiological understanding of MS include the recognition of B-cells as key mediators of subacute inflammatory tissue damage (leading to a whole new line of potent anti-CD20 disease-modifying treatments) [6], local innate immune responses that drive the chronic neurodegeneration of progressive MS [7,8], and cognitive impairment manifesting as a potential early manifestation of the disease [9]. Epstein–Barr virus (EBV) infection has long been epidemiologically linked to MS, but a true causative association was difficult to prove. However, in early 2022, a study by Bjornevik and co-workers, using data from more than 10 million military recruits in the United States of America monitored over a 20-year period, found that EBV seropositivity preceded MS onset in virtually all patients. The risk of MS increased with a 32-fold factor after infection with EBV but was not increased after infection with other, similarly transmitted viruses. Moreover, serum levels of neurofilament light chain (NfL), a well-established biomarker of neurodegeneration, increased exclusively after EBV seroconversion. These findings could not be explained by any other known risk factor and make a very convincing case for EBV as the leading cause of MS [10]. Interestingly, Lanz and colleagues subsequently reported a high-affinity and pathologically relevant molecular mimicry between the EBV transcription factor EBV nuclear antigen 1 and a post-translationally modified glial cell adhesion molecule (called GlialCAM) in approximately 25% of people with MS, providing a mechanistic link between the viral infection and the B-cell mediated targeting of the central nervous system (CNS) [11]. These novel insights might pave the way for upcoming antiviral and/or vaccination strategies aimed at disease modification or even curation/prevention.

In the classic teaching of MS, a clear distinction was made between the three classic clinical phenotypes: relapsing-remitting, secondary progressive, and primary progressive disease. Whereas recurrent autoimmune inflammatory attacks directed against the CNS’s myelin were considered the dominant force behind the relapsing phase, progressive MS was attributed to a subsequent, yet less well understood, slowly continuous neurodegenerative process. Recent literature has challenged this rather linear view by revealing that gradual disease progression independent of (inflammatory) relapse activity—for which the acronym PIRA has been coined—is an important contributor to global disability accumulation in patients with a relapsing phenotype as well [12,13,14], including in the very early stages [15,16]. In addition, it has become clear that accelerated brain volume loss (BVL) and cognitive decline, two features previously mainly associated with progressive MS, can already be present at the time of diagnosis of relapsing-remitting MS or even before [17,18,19,20]. Thus, the current understanding of MS pathology now relies on a concept in which inflammatory and neurodegenerative mechanisms concurrently evolve throughout the disease course, regardless of historic clinical phenotyping. Recently, and perhaps more importantly, it has also been recognized that progressive MS might be driven by a different form of inflammation which is restricted to the CNS, occurring behind a closed blood–brain barrier and characterized by submeningeal collections of lymphoid cells organized as follicle-like structures and chronic enlarging demyelinating lesions (also termed smoldering lesions) with an expanding border of iron-loaded activated microglia [21,22]. Smoldering lesions can be visualized in humans by susceptibility-weighted magnetic resonance imaging as paramagnetic rim lesions, which seem to correlate well with concurrent and forthcoming clinical disability and BVL [23,24,25,26]. In addition, recent studies have associated such chronically expanding lesions with toxic sodium accumulation and neurofilament release in the CNS [27,28], both of which are part of the neurodegenerative cascade in MS [29]. Microglial targeting has become a new treatment strategy in both progressive and relapsing diseases, with several agents currently being tested in phase III trials [30].

In conjunction with pharmacological approaches, alternative treatment strategies may also slow down disease progression and/or improve a patient’s daily functioning. As an example, although disease-modifying treatments used for MS have limited effects on cognitive functioning, the literature suggests that cognitive, physical, and dual-task rehabilitation can improve cognitive performance, potentially in a synergistic manner [31,32,33,34,35], and may even have neuroprotective properties, including remyelination via the stimulation of both new and surviving oligodendrocytes [36,37,38]. In addition, novel developments in transcranial electrical stimulation have demonstrated restored working memory capacity in healthy elderly people by imposing specific electrical stimulation in specific brain regions [39]. Current immunomodulatory MS drugs have been relatively successful in preventing new inflammatory episodes, but restorative and/or neuroprotective treatment is still lacking and undeniably represents an unfulfilled scientific goal. Understanding and managing the double-agent role of microglia will be a key element towards true neuroprotection in MS, as we have seen that a detrimental activation pattern can trigger neurodegeneration while these cells also have essential reparative properties such as clearing debris and regulating myelin growth [40]. Interestingly, we have started 2023 with the hopeful message that neural stem cell transplantation was feasible, safe, and associated with radiological/biochemical signs of neuroprotection [41].

Biomarkers, capable of detecting small treatment effects, could significantly accelerate the development of drugs and other treatments and ameliorate the outcome of therapeutic decisions. These biomarkers could assess brain structure (e.g., brain age) brain functioning, serum, or CSF biomarkers [42,43,44]. It is important to note that although it is modish to state that artificial intelligence (AI) will enable the development of multimodal biomarkers, several challenges remain. The key to success lies within the carefully curated dataset rather than in novel insights uncovered by an AI algorithm. For a more extensive discussion, we refer to a recent controversial section in the *Multiple Sclerosis Journal* [45,46]. It has also been suggested that digital app-based biomarkers could reduce treatment costs through earlier disease progression detection [47].

Finally, it is worth noting that digital teleconsultations, catalyzed by the recent coronavirus 2019 pandemic, have the potential to reduce health costs and improve access to neurological care facilities. This was illustrated by Kadel and co-workers, who set up a tele-video consultation system that avoided 73% of transfers for neurosurgical emergency management in Italy [48]. Telehealth monitoring is also feasible and promising in the longitudinal follow-up of people with neurodegenerative diseases. Sadeghi et al. achieved a rate of 87% successfully completed telehealth consultations over a follow-up period of 12 months in patients with MS [49], whereas Beck et al. reported similar outcomes in individuals with Parkinson’s disease [50]. However, although teleconsultation is expected to consolidate its place in the neurology clinic of the future, we must not neglect recent signals of digital medicine aggravating existing social disparities in healthcare access in somewhat unexpected and contradictory ways [51,52,53].

In conclusion, global care for chronic neurodegenerative disorders is expensive and suffers from logistic resource limitations. These issues likely will only increase in the future due to an aging population. Using the example of MS, we stress the importance of improving our basic pathophysiological understanding to develop more effective drug treatment and disease prevention strategies. Non-pharmacological interventions, such as physical rehabilitation and electrical stimulation techniques, may be worth exploring as potential add-on treatments. We further contend that multimodal biomarkers will help us stratify patients and install precision medicine. Finally, we stress the potential of telehealth to increase accessibility and further reduce clinical care costs.
